# Comprehensive Analysis of Key m6A Modification Related Genes and Immune Infiltrates in Human Aortic Dissection

**DOI:** 10.3389/fcvm.2022.831561

**Published:** 2022-03-14

**Authors:** Fanxing Yin, Hao Zhang, Panpan Guo, Yihao Wu, Xinya Zhao, Fangjun Li, Ce Bian, Chen Chen, Yanshuo Han, Kun Liu

**Affiliations:** ^1^School of Life and Pharmaceutical Sciences, Dalian University of Technology, Dalian, China; ^2^Department of Cardiovascular Surgery, The General Hospital of the PLA Rocket Force, Beijing Normal University, Beijing, China; ^3^School of Biomedical Sciences, The University of Queensland, Brisbane, QLD, Australia; ^4^Department of Cardiac Surgery, Affiliated Hospital of Guizhou Medical University, Guiyang, China

**Keywords:** acute aortic dissection, m6A, bioinformatics, immune infiltration, hub genes

## Abstract

**Objective:**

To identify the feature of N6-methyladenosine (m6A) methylation modification genes in acute aortic dissection (AAD) and explore their relationships with immune infiltration.

**Methods:**

The GSE52093 dataset including gene expression data from patients with AAD and healthy controls was downloaded from Gene Expression Omnibus (GEO) database in order to obtain the differentially expressed genes (DEGs). The differentially methylated m6A genes were obtained from the GSE147027 dataset. The differentially expressed m6A-related genes were obtained based on the intersection results. Meanwhile, the protein-protein interaction (PPI) network of differentially expressed m6A-related genes was constructed, and hub genes with close relationships in the network were selected. Later, hub genes were verified by using the GSE153434 dataset. Thereafter, the relationships between these genes and immune cells infiltration were analyzed.

**Results:**

A total of 279 differentially expressed m6A-related genes were identified in the GSE52093 and GSE147027 datasets. Among them, 94 genes were up-regulated in aortic dissection (AD), while the remaining 185 were down-regulated. As indicated by Gene Ontology (GO) functional enrichment and Kyoto Encyclopedia of Genes and Genomes (KEGG) pathway enrichment analyses, these genes were mainly associated with extracellular matrix (ECM) and smooth muscle cells (SMCs). The seven hub genes, namely, DDX17, CTGF, FLNA, SPP1, MYH11, ITGA5 and CACNA1C, were all confirmed as the potential biomarkers for AD. According to immune infiltration analysis, it was found that hub genes were related to some immune cells. For instance, DDX17, FLNA and MYH11 were correlated with Macrophages M2.

**Conclusion:**

Our study identifies hub genes of AD that may serve as the potential biomarkers, illustrates of the molecular mechanism of AD, and provides support for subsequent research and treatment development.

## Introduction

Aortic dissection (AD) represents a pathological change that separates the true and false lumens of the aorta. Due to rupture of the aortic intima or disease in the media elastic fibrous layer caused by various etiologies, blood in the aortic lumen enters the media through the torn intima, as a result, the media will tear and thus a dual-lumen aorta is formed (1). AD that develops within 2 weeks is defined as AAD, while AD that develops over 2 weeks later is defined as subacute AD or chronic AD (2). AAD is associated with the highest mortality rate. The annual incidence of AAD in the general population is 3.5–6 cases per 100,000 people, but it can increase to 10 cases or more in the elderly population (3). The chance of survival can be greatly improved when AAD is detected early and treated promptly (4). At present, AAD is mainly treated by open surgical repair, which requires a standardized treatment center and a high level of technology. Meanwhile, thoracic endovascular aortic repair (TEVAR) has also evolved to treat AD (5). In recent years, with the development of monitoring technology to detect AAD markers and the update of treatment modalities, the survival rate of AAD patients shows an increasing trend, but its overall survival (OS) rate remains poor. Moreover, due to the restricted geographical, economic and technical conditions in some underdeveloped areas, not all patients can receive treatment in time (6). Therefore, scholars should pay more attention to the pathogenesis of AD. In this regard, it is necessary to develop new subsequent treatment approaches through research on AAD at cellular and gene levels.

Recently, with the development and rise of technologies such as high-throughput sequencing and DNA microarray, the research on the potential molecular mechanisms of AAD has been further promoted. Nowadays, the pathogenesis of AAD is still unclear. Treatment time represents the greatest challenge in the treatment of AD, and many patients still die before surgery (4). Some genes are related to the occurrence and development of AAD. Therefore, it is an extremely urgent task to identify genes that can serve as the hallmarks in the occurrence and development of AAD. In the past, AD was suggested to be associated with single gene mutation, but numerous scholars try to investigate AD from the perspective of single nucleotide polymorphism (SNP) at present. Some genes related to AAD have been identified, such as MYH11, TGFBR1 and MMP-8. The polymorphisms of these genes are significantly related to the susceptibility of AD (7–9). Current research mainly focuses on the potential mechanism between epigenetic modification and AD (10). A variety of epigenetic modifications have been detected, including the most commonly seen DNA methylation, histone methylation and acetylation, and chromosomal remodeling (11, 12). Of course, there are also modification models at the RNA level (13). Briefly, it is important to find more hub genes for AAD, which may help the development of AAD diagnosis and treatment. Although it is challenging to find the hub genes through experiments, especially the genes with epigenetic modification, it is more feasible to explore with bioinformatics methods.

Over 160 types of chemical modifications have been identified in RNA, including m6A, N1-methyladenosine (m1A), 5-methylcytosine (m5C), and N7-methylguanosine (m7G) (13). Among them, m6A is the most prevalent and abundant type of internal RNA modification in eukaryotic cells at the post-transcriptional level (14). m6A is the methylation of the adenosine at the nitrogen-6 position (15), and its processing centers are writers, readers, and erasers. Of them, writers and erasers are responsible for catalyzing and removing m6A, respectively (16). For instance, METTL3 is a writer whose down-regulation contributes to the decreased m6A modification of vascular smooth muscle cells (SMCs)-specific markers, thus leading to the reductions in their mRNA and protein expression (17). In terms of vascular diseases, m6A is related to atherosclerosis (AS), ischemic heart disease, and heart failure (HF). With regard to aortic diseases, m6A is involved in the occurrence and development of aortic aneurysms (14). However, the researches on m6A and AD are insufficient. Therefore, we attempted to discover some potential mechanisms of m6A in AD.

This study aimed to explore the possible molecular mechanisms of m6A in AD and provide certain theoretical basis for the development of AD etiology from the perspective of m6A. In this study, AD and m6A-related expression profile data were downloaded from the GEO database for analysis. First of all, the differentially expressed m6A-related genes between AD patients and normal tissues were detected. Thereafter, GO and KEGG analyses were conducted on the selected genes, and then a PPI network was established to identify hub genes related to m6A in AD. Afterwards, the immune infiltration in AD samples and normal tissue samples was analyzed. Finally, information regarding the immune infiltration, including correlation analysis, was obtained. Our research can provide clues for further research on the molecular mechanisms of epigenetic changes in AD patients and help identify new m6A methylation markers.

## Materials and Methods

### Data Sources

The gene expression dataset was downloaded from the GEO database (https://www.ncbi.nlm.nih.gov/geo/) for analysis. The database was searched for a series of studies on human AAD. Then, four gene expression datasets were screened (GSE52093, GSE147027, GSE92427 and GSE153434). Among them, GSE52093 included gene expression data from patients with AAD (*n* = 7) and healthy controls (*n* = 5). After preprocessing, data of two unqualified AD samples were eliminated (GSM1259277 and GSM1259278). GSE147027 included m6A RNA methylome analyses between AAD (*n* = 2) and normal human aorta (*n* = 2). In GSE92427, the microRNA (miRNA) microarray was used for expression profile analysis from 16 plasma samples, including AAD patients (*n* = 8) and healthy subjects (*n* = 8). GSE153434 included gene expression data of dissected aorta from AAD patients (*n* = 10) and normal controls (*n* = 10). All data were free to use, and this study did not involve any experiments on humans or animals.

### Weighted Gene Co-expression Network Analysis

The genes with similar expression behavior in GSE52093 were divided into different modules. This study adopted the R software package WGCNA for constructing a gene co-expression network. After determining the soft threshold, the network was developed. Module-trait relationships were calculated based on a Pearson correlation. The modules with *P* < 0.05 were regarded as significantly correlated modules.

### Data Collection and Screening of Differentially Expressed m6A-Related Genes

Normalization of the data was performed with the R packages “sva” and “limma.” Differential expression analysis of genes in different modules between AAD and healthy controls was performed using the R software package “impute” and “limma.” Genes with adjusted *P* < 0.05 and |*logFC*| ≥ 1 were selected as differentially expressed genes (DEGs). The differentially methylated m6A genes between AAD and normal human aorta in GSE147027 were downloaded from the GEO database, and adjusted *P* < 0.05 was defined as significant. Thereafter, DEGs were intersected with these differentially methylated m6A genes to obtain the differentially expressed m6A-related genes. These genes were further analyzed, including the chromosomes where they were located and their relations with differentially expressed miRNAs (DEmiRs). DEmiRs were selected upon the thresholds of adjusted *P* < 0.05 and |*logFC*| ≥ 1 by the R software package “impute” and “limma.” The mRNAs that showed interactive relationship with DEmiRs from three databases (miRDB, RTarBase and starBase) were found and intersected with the differentially expressed m6A-related genes. Based on the overlapped mRNAs and DEmiRs, a network of DEmiRs-m6A genes pairs was constructed.

### GO and KEGG Pathway Analysis

GO and KEGG pathway enrichment analyses of differentially expressed m6A-related genes were performed using the Database for Annotation, Visualization and Integrated Discovery (DAVID) tools (https://david.ncifcrf.gov/). GO functional annotation classifies gene functions into biological processes (BP), cellular components (CC) and molecular functions (MF). *FDR* < 0.05 and gene counts >5 were considered statistically significant.

### PPI Network Construction and Hub Gene Identification

The Search Tool for the Retrieval of Interacting Genes (STRING) database (https://www.string-db.org) was employed to construct a PPI network for differentially expressed m6A-related genes. Then, the PPI pairs were extracted using a combined score of >0.55. Subsequently, the PPI network was visualized by Cytoscape software. According to the maximal clique centrality (MCC) method, CytoHubba was employed to calculate the degree of each protein node. MCODE, a plugin in Cytoscape, was utilized to analyze the sub-networks (highly interconnected regions) in the PPI network. Later, genes existing in the sub-networks with the top 10 degrees in the whole PPI network were considered as hub genes. ROC monofactor analysis was performed to evaluate the diagnostic value of m6A-related biomarkers in AD. Then, A LASSO model was established to identify the hub genes by “glmnet” package. In order to ensure more accurate results, GSE153434 was applied to verify the differential expression of hub genes.

### The Relationship Analysis Between Differentially Expressed m6A Regulatory Genes and Hub Genes

HAKAI, METTL3, METTL14, METTL16, RBM15, RBM15B, WTAP, VIRMA, ZC3H13 and ZCZHC4 are m6A writers. ALKBH5 and FTO are m6A erasers. HNRNPA2B1, HNRNPC, IGF2BP1, IGF2BP2, IGF2BP3, YTHDC1, YTHDC2, YTHDF1, YTHDF2 and YTHDF3 are m6A readers. The above 22 genes are regarded as common m6A methylation regulators. Thereafter, these m6A regulators which had significant difference were considered as differentially expressed m6A regulatory genes (DEMRGs). The target genes of DEMRGs were predicted with the m6a2Target database (http://m6a2target.canceromics.org). Then, the hub genes, which were target genes, were found.

### Analysis of Immune Cell Infiltration

CIBERSORTx can obtain the molecular characteristics of different cell types from the signature gene file, and extract the transcriptome of a single cell type from mass data, with no need to separate individual cells (18, 19). The gene expression matrix of GSE153434 was uploaded to CIBERSORTx (https://cibersortx.stanford.edu). Meanwhile, the leukocyte signature matrix (LM22), which can distinguish 22 human hematopoietic cell phenotypes, was selected as the signature gene file. Afterwards, data were processed through CIBERSORTx, and the results of immune cell infiltration were output. Then, the contents of diverse immune cell types in each sample were visualized according to the results.

### Analysis of the Correlations Between Hub Genes and Immune Cells

To further analyze the mechanism between m6A-related genes and immune cells during the development of AAD, we used the relative proportion of immune cells to explore the correlation between immune cells. The absolute ratio data of immune cells was performed to analyze the relationship between hub genes and immune cells. Pearson correlation coefficient was used to identify interaction relationships.

## Results

**Figure 1:**
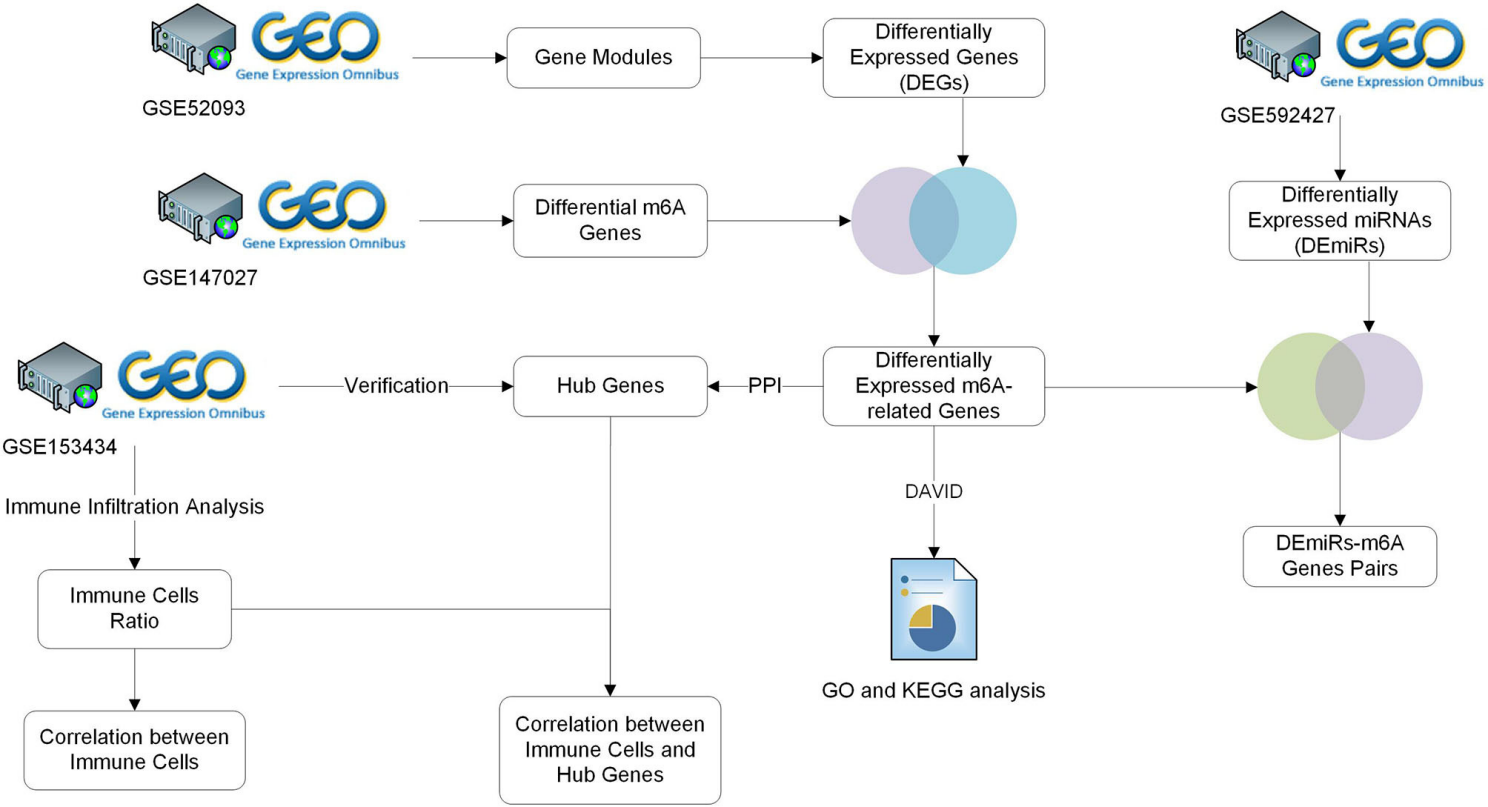
Study workflow. GEO, Gene Expression Omnibus; GO, Gene Ontology; KEGG, Kyoto Encyclopedia of Genes and Genomes; TARGET, Therapeutically Applicable Research to Generate Effective Treatments.

In the present study, the biological characteristics of DEGs and differential m6A methylation genes were identified by integrated bioinformatics analysis. The overall workflow of this study is displayed in Figure 1. The differentially expressed m6A-related genes in data pre-processing was shown in Supplementary File 1.

### WGCNA

**Figure 2:**
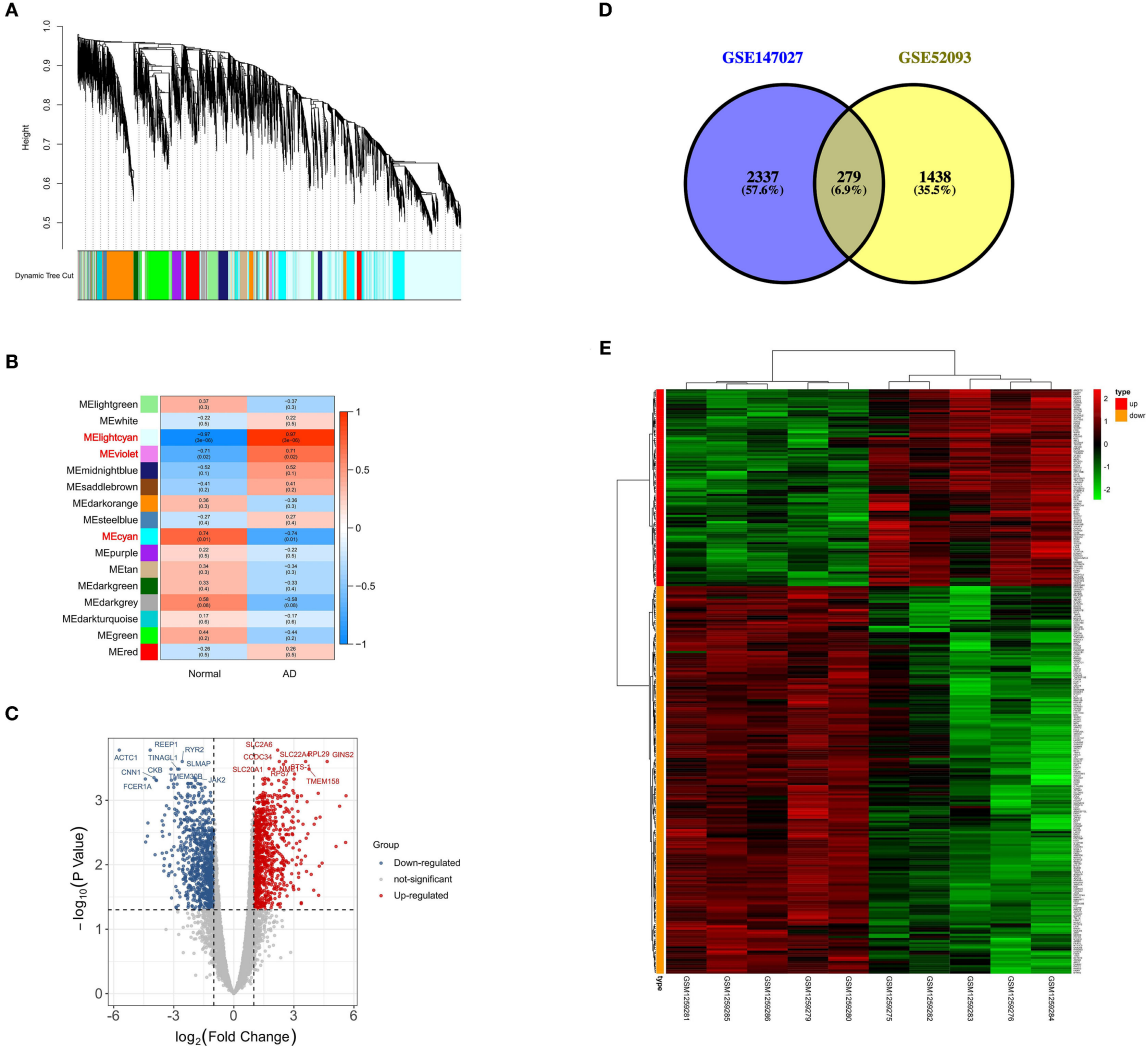
Screening of differentially expressed m6A-related genes. **(A)** Gene dendrogram and module colors in GSE52093; **(B)** Module-trait relationship, red represents positive correlation, and blue represents negative correlation; **(C)** Volcano plot of DEGs, red represents up-regulated genes and blue represents down-regulated genes; **(D)** Venn plot of differentially expressed m6A-related genes from two datasets in acute type A aortic dissection; **(E)** Heatmap of the expression patterns of 279 differentially expressed m6A-related genes.

In this study, β = 6 was selected as the soft thresholding power to ensure that the network obeyed the scale-free criteria. Gene dendrogram and module colors were presented in Figure 2A. The established network included 16 modules. Genes were categorized based on module-trait relationships. There were three significantly high correlated modules, namely MElightcyan, MEviolet and MEcyan (Figure 2B), and a total of 4,566 genes were obtained in these modules. The module-trait relationship of MElightcyan was −0.97 in normal samples and 0.97 in AAD samples, while that of MEviolet was −0.71 in normal samples and 0.71 in AAD samples, and that of MEcyan was 0.74 in normal samples and −0.74 in AAD samples.

### Identification of Differentially Expressed m6A-Related Genes

Based on the genes obtained in differential modules, the distinct expression analysis of genes between AAD tissues and controls was conducted, as shown in the volcano plot of DEGs (Figure 2C, Supplementary File 2). Altogether 2616 differentially methylated m6A genes were selected from 3721 differential methylation sites in GSE147027 (Supplementary File 3). Thereafter, 1717 DEGs were intersected with 2616 differentially methylated m6A genes to obtain 279 differentially expressed m6A-related genes (Figure 2D). The expression patterns of these genes were presented in Figure 2E.

**Figure 3:**
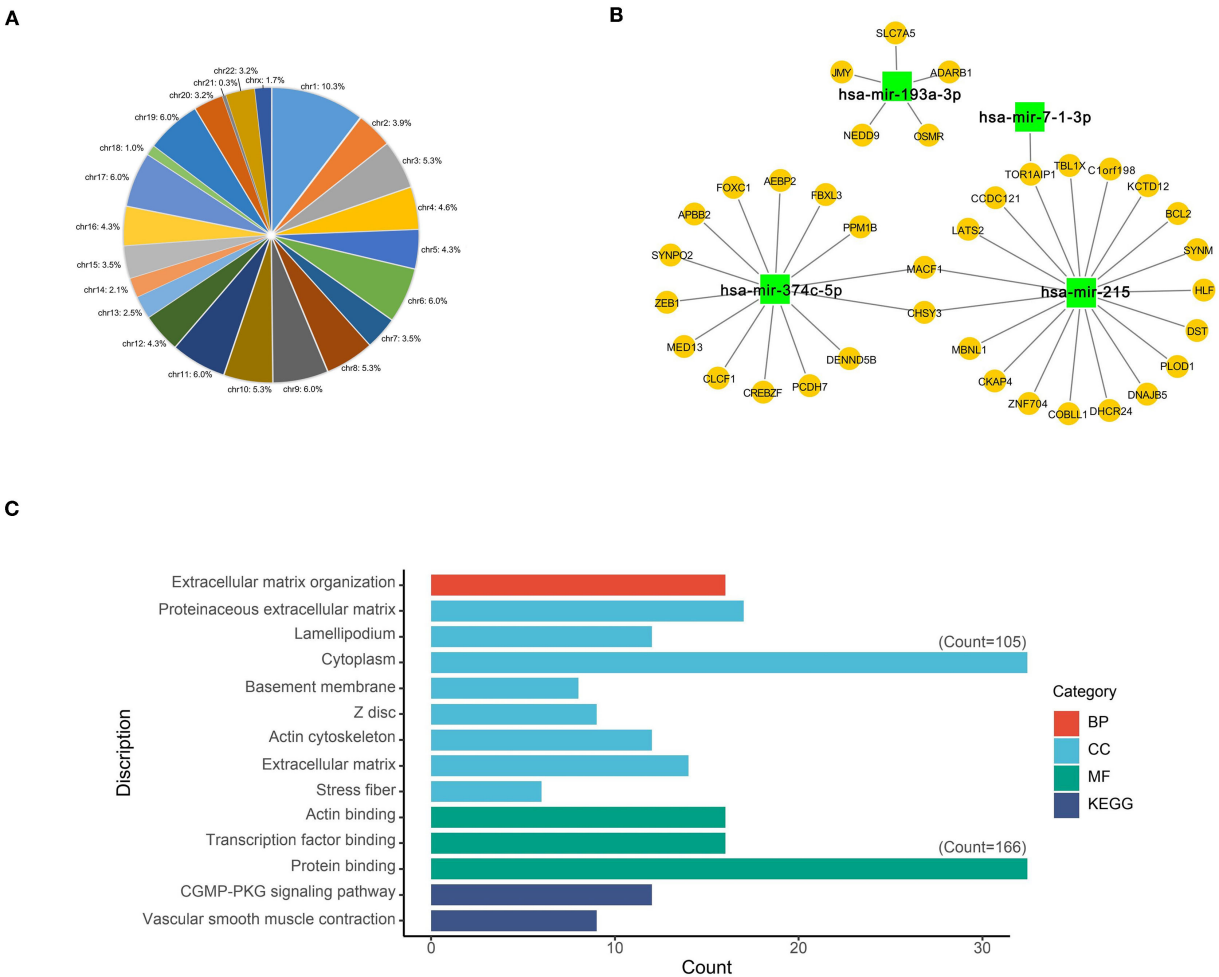
Functional characteristics of differentially expressed m6A-related genes. **(A)** Chromosome location of differentially expressed m6A-related genes; **(B)** Network of DEmiRs and differentially expressed m6A-related genes; **(C)** The main GO and KEGG enrichment results of differentially expressed m6A-related genes based on David database.

As a result, most of the differentially expressed m6A-related genes were concentrated at the chr1 position (Figure 3A). Four DEmiRs were detected in GSE92427 and incorporated to establish a network that involved 36 overlapping mRNAs between the DEmiRs-linked mRNAs and the differentially expressed m6A-related genes (Figure 3B, Supplementary File 4).

### Functional Enrichment Analyses of Differentially Expressed m6A-Related Genes

GO and KEGG enrichment analyses of differentially expressed m6A-related genes were performed using the DAVID, so as to interpret the clustering results. As indicated by GO analysis, these genes were mainly involved in CCs, including proteinaceous extracellular matrix, lamellipodium, cytoplasm, basement membrane, Z disc, actin cytoskeleton, extracellular matrix and stress fiber. With regard to MFs, these genes were mainly enriched in actin binding, transcription factor binding and protein binding. In terms of BPs, these genes were mostly enriched in the regulation of BP such as extracellular matrix organization. Moreover, according to KEGG pathway enrichment analysis, these differentially expressed m6A-related genes were mainly enriched in two pathways, namely, cGMP-PKG signaling pathway and Vascular smooth muscle contraction (Figure 3C, Supplementary File 5).

### PPI Network Construction and Hub Gene Identification

**Figure 4:**
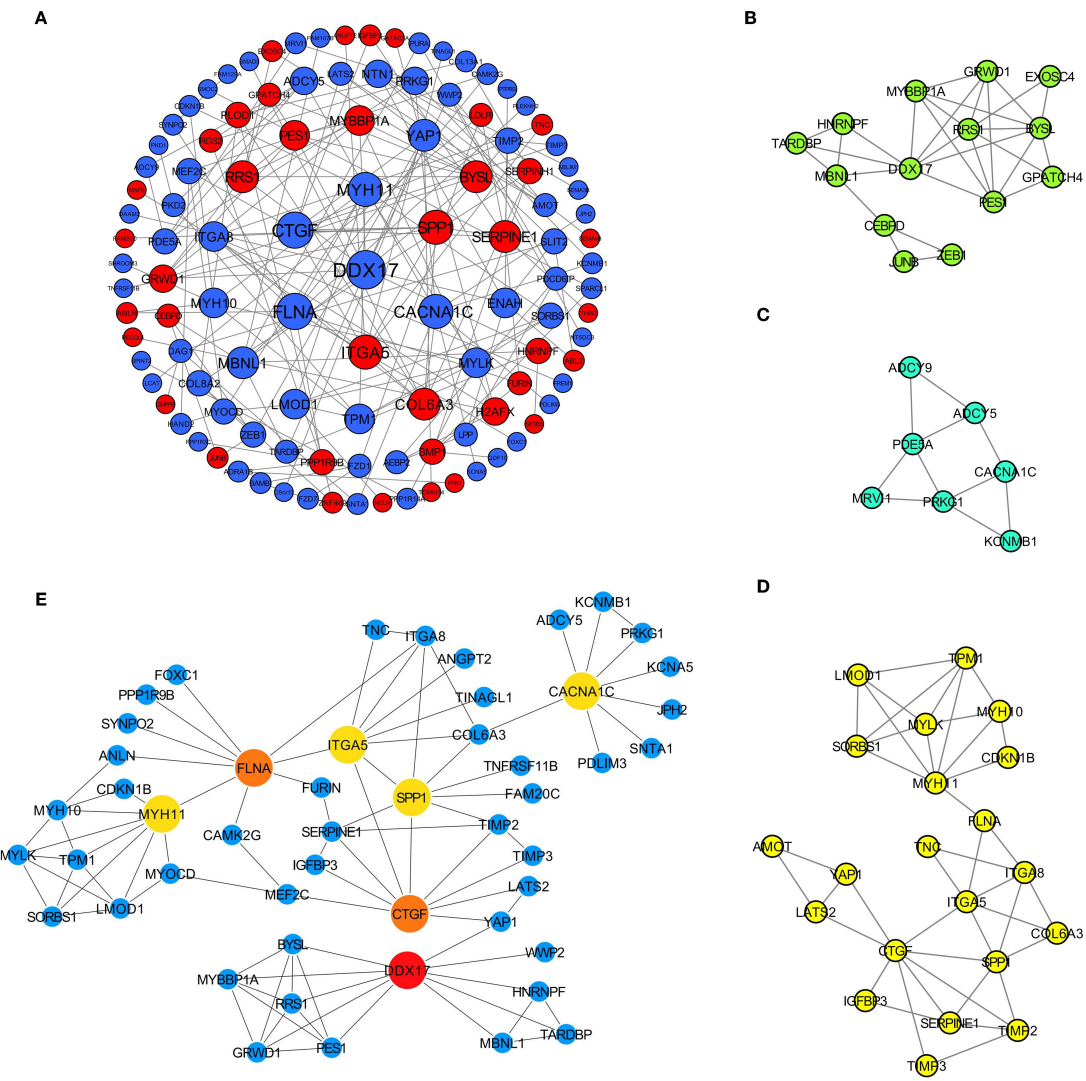
Identification of hub differentially expressed m6A-related genes for AD. **(A)** PPI network of 279 differentially expressed m6A-related genes based on STRING database; **(B–D)** Three sub-networks of whole PPI network; **(E)** Interaction relationships of 7 hub genes.

Afterwards, the differentially expressed m6A-related genes were uploaded to the STRING online database to analyze the protein interactions, and the PPI network was constructed using Cytoscape software (Figure 4A). The highly interacted genes were selected using CytoHubba, and three sub-networks were constructed by MCODE (Figures 4B–D). Then, seven differentially expressed m6A-related genes were obtained as the hub genes, which existed in the sub-networks simultaneously, with the top 10 degrees in the whole PPI network (Figure 4E).

**Figure 5:**
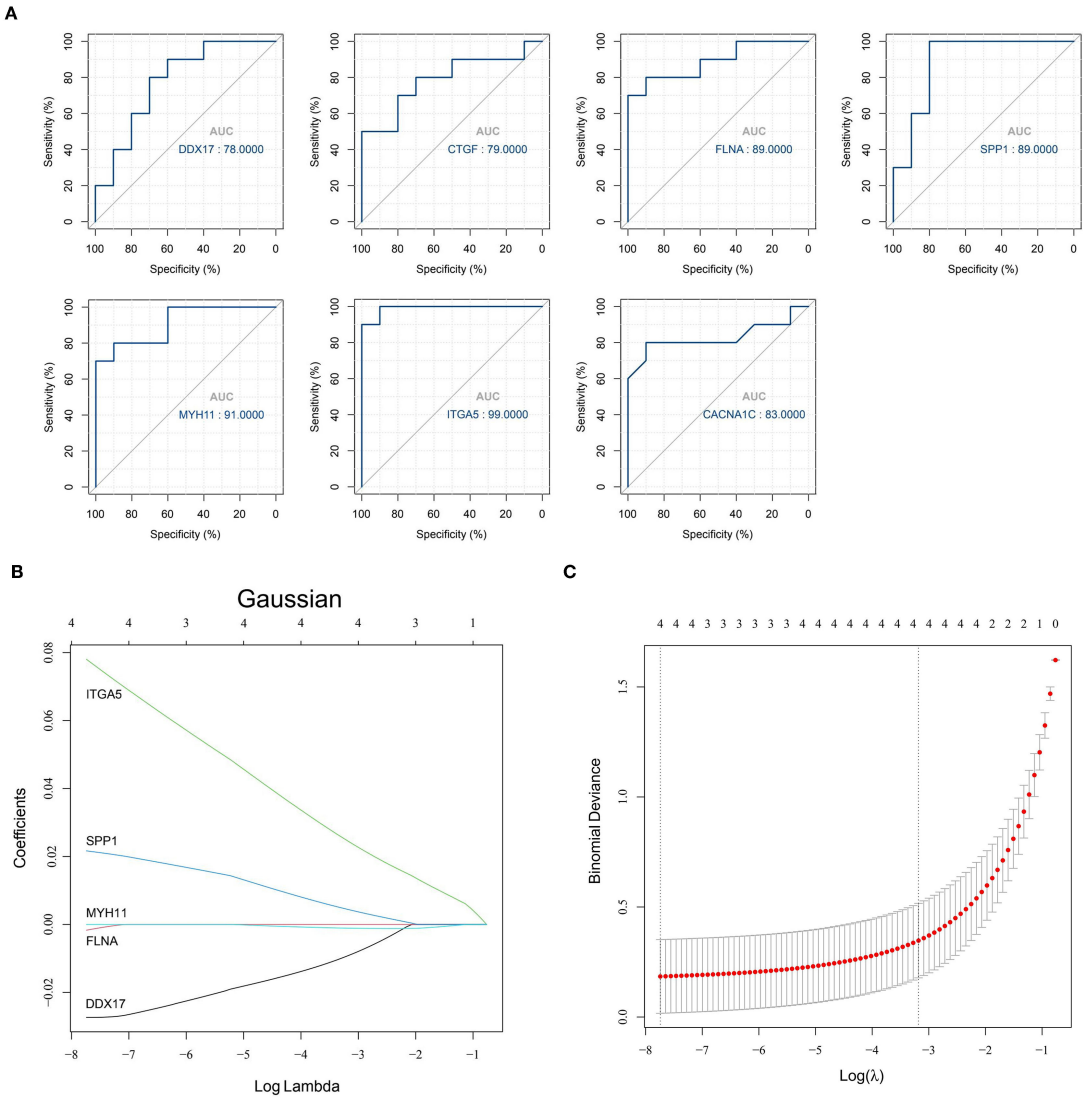
Results of the models for predicting AD. **(A)** ROC curve analysis of hub genes; **(B,C)** LASSO model of hub genes.

**Figure 6:**
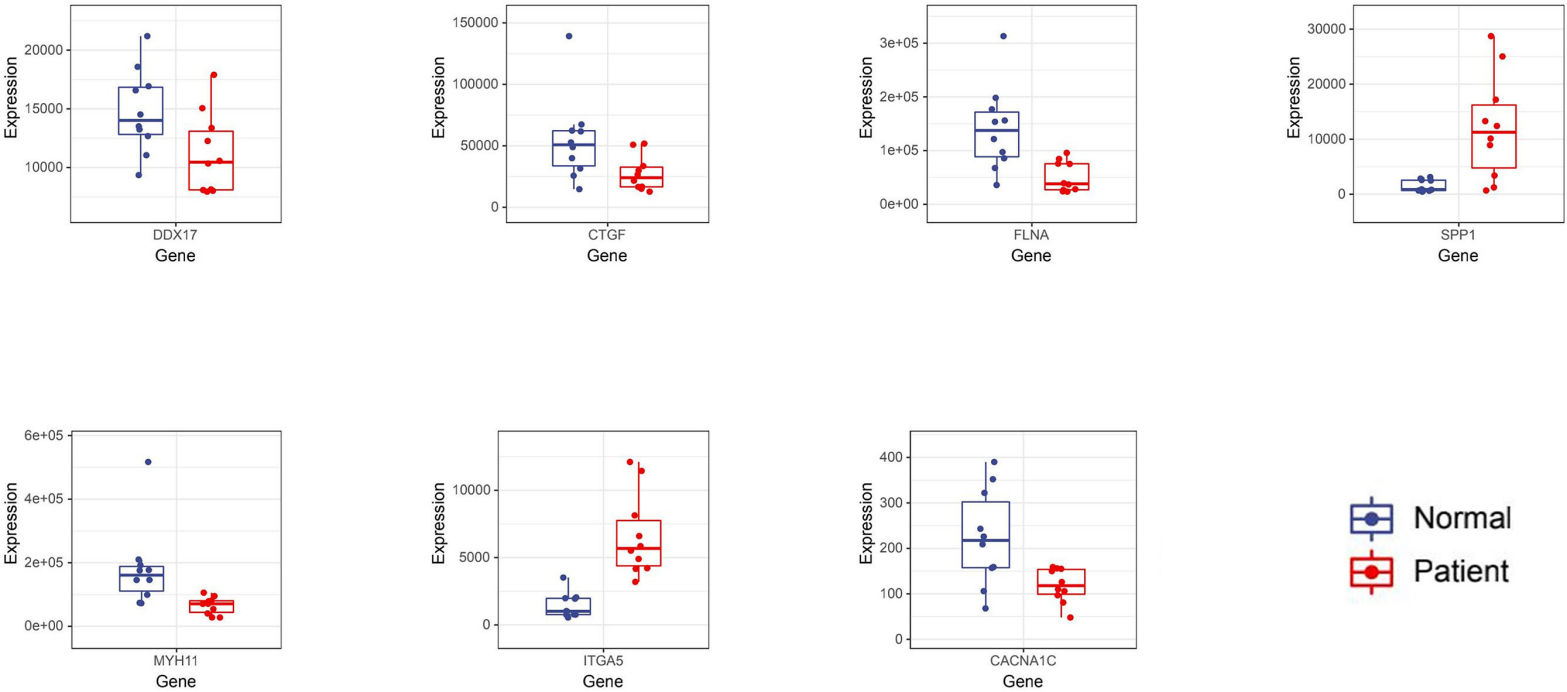
The differential expression of 7 hub genes was verified in GSE153434.

The obtained hub genes included DEAD-box helicase 17 (DDX17), connective tissue growth factor (CTGF) that was also named cellular communication network factor 2 (CCN2), filamin A (FLNA), secreted phosphoprotein 1 (SPP1), myosin heavy chain 11 (MYH11), integrin subunit alpha 5 (ITGA5), and calcium voltage-gated channel subunit alpha1 C (CACNA1C). Of them, SPP1 and ITGA5 were up-regulated in AD, whereas the others were down-regulated. A network of hub genes and other genes that directly interacted with these hub genes was constructed. Later, ROC monofactor analysis was performed to evaluate the diagnostic value of the seven AD biomarkers. Consequently, the diagnostic accuracy (AUC) of DDX17, CTGF, FLNA, SPP1, MYH11, ITGA5 and CACNA1C for AD was 78, 79, 89, 89, 91, 99 and 83, respectively, in the GSE52093 dataset (Figure 5A). Hub genes also had good diagnostic value in GSE153434 which was selected as the test set (Supplementary File 6). Five genes (DDX17, FLNA, SPP1, MYH11 and ITGA5) were identified to construct the gene signature through LASSO regression analysis (Figures 5B,C). Seven of the above hub genes were also verified to be significant in GSE153434 (Figure 6).

### The Relationship Between DEMRGs and Hub Genes

**Figure 7:**
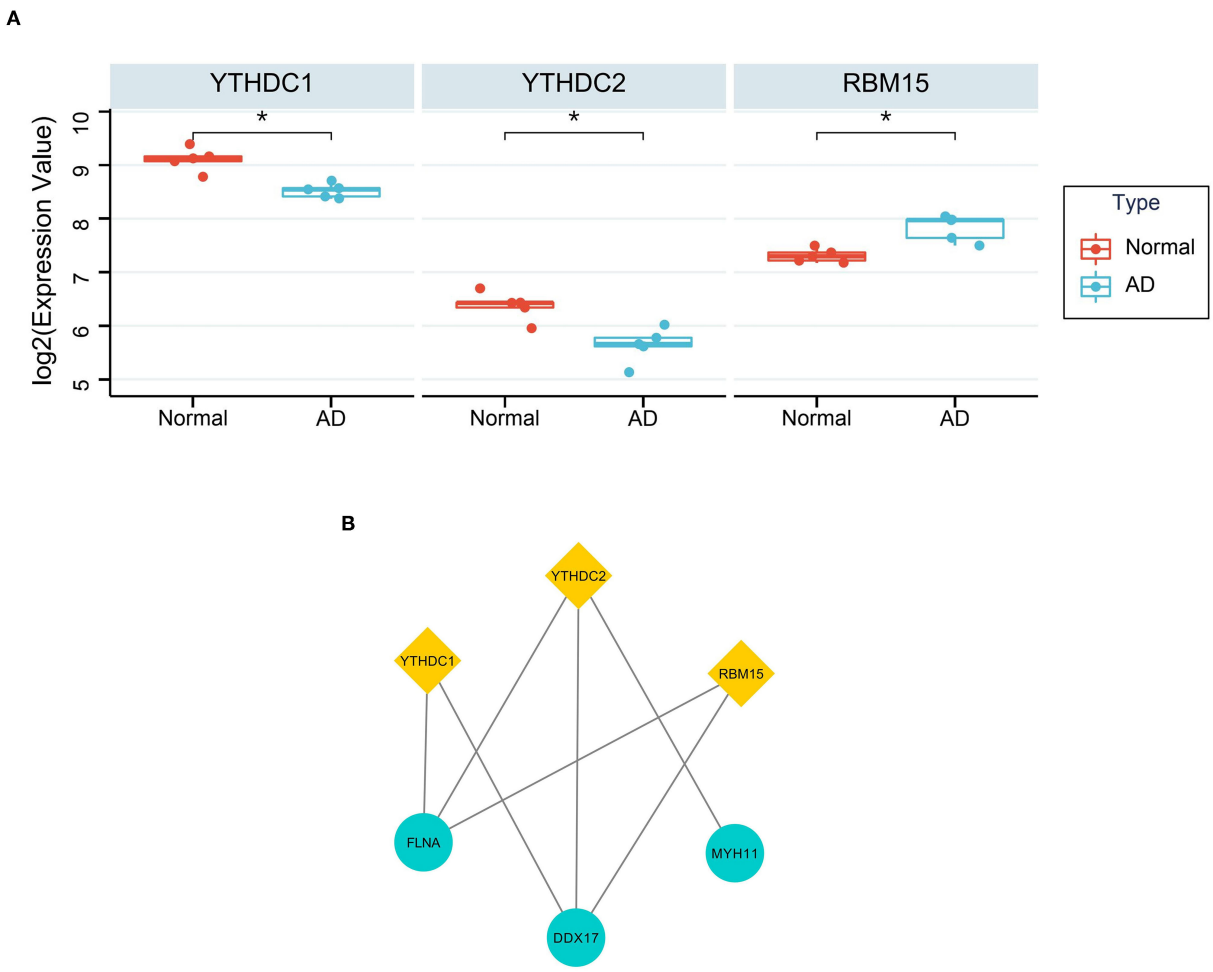
The relationship between DEMRGs and hub genes. **(A)** The differential expression of DEMRGs. *Represented *p* < 0.05; **(B)** The target genes of DEMRGs in hub genes.

YTHDC1 (*P* = 0.021 and *logFC* = −0.583), YTHDC2 (*P* = 0.026 and *logFC* = −0.730) and RBM15 (*P* = 0.037 and *logFC* = 0.516) were considered as DEMRGs (Figure 7A). Among the hub genes, DDX17, FLNA and MYH11 were the target genes of DEMRGs. DDX17 and FLNA were the common target genes of the three DEMRGs (Figure 7B).

### Results of Immune Cell Infiltration

**Figure 8:**
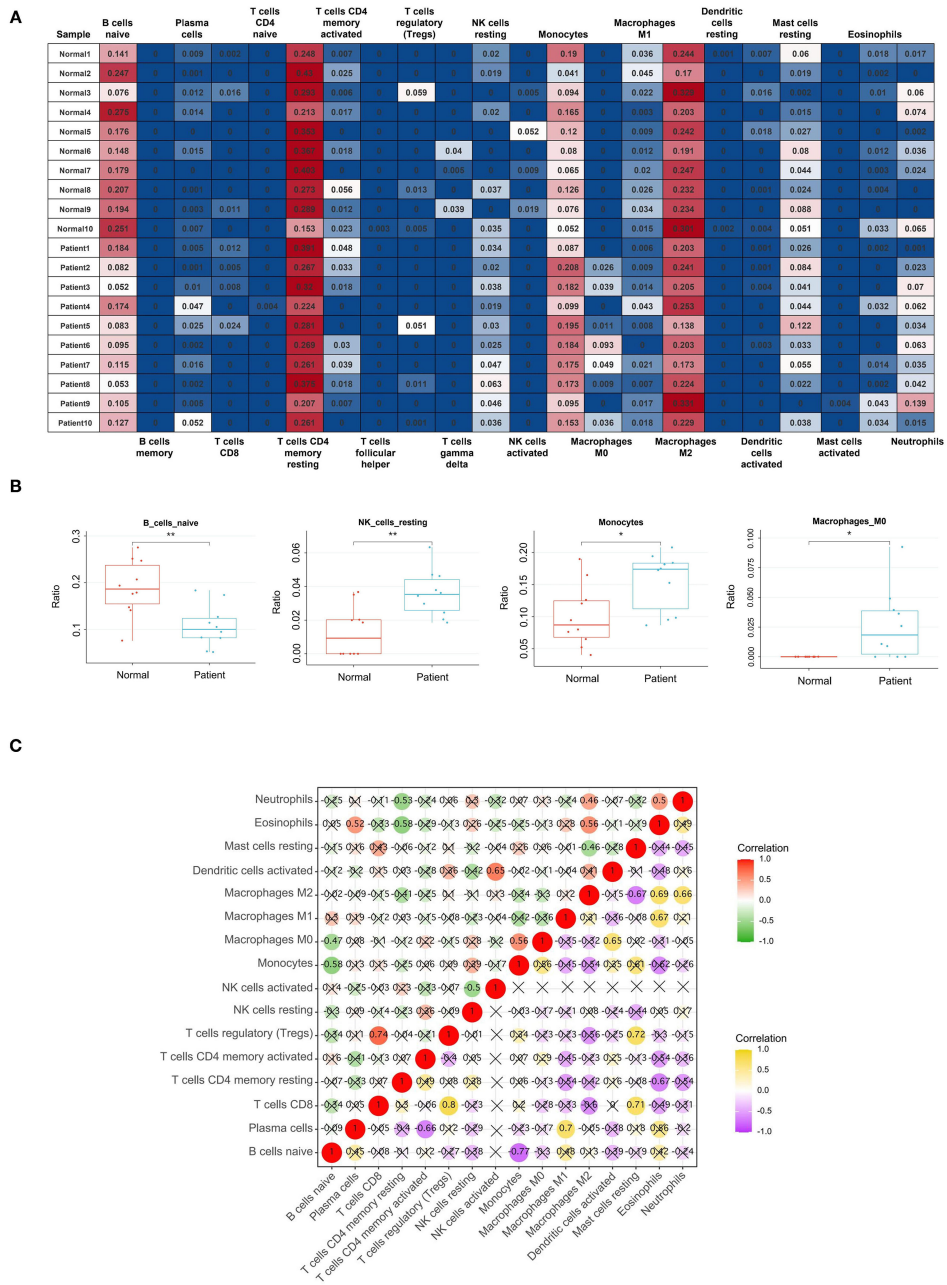
Results of Immune Cell Infiltration analysis. **(A)** The percentages of immune cells in each sample of GSE153434; **(B)** The content of B cells naive, NK cells resting, Monocytes and Macrophages M0 in normal aortic samples and AAD samples. *Represented *p* < 0.05. **Represented *p* < 0.01; **(C)** The relationships of immune cells in all samples and in AAD samples only, the correlation analysis of immune cells of all samples is shown in red and green, with red representing positive correlation and green representing negative correlation. The correlation analysis of immune cells of only AAD samples is represented by purple and yellow, yellow represents positive correlation, and purple represents negative correlation.

After data normalization, the GSE153434 dataset was uploaded to the CIBERSORTx online database to analyze immune cell infiltration. Later, the percentages of immune cells in 10 AAD samples and 10 normal aortic samples were calculated by CIBERSORTx (Figure 8A). The relative abundances of B cells naive, NK cells resting, Monocytes and Macrophages M0 were significantly different between normal aortic samples and AAD samples (Figure 8B).

After filtering out the immune cell types that were not present (0 value in more than 80% of samples), the remaining 16 types of immune cells were subject to Pearson correlation analysis, including the relationships of immune cells in all samples and in AAD samples only (Figure 8C). For example, in all samples, NK cells activated were significantly positively correlated with Dendritic cells activated (r = 0.65), and B cells naive were markedly negatively related to Monocytes (r = −0.58). Macrophages M2 were significantly correlated with Eosinophils (r = 0.69) and Neutrophils (r = 0.66) in AAD samples.

**Figure 9:**
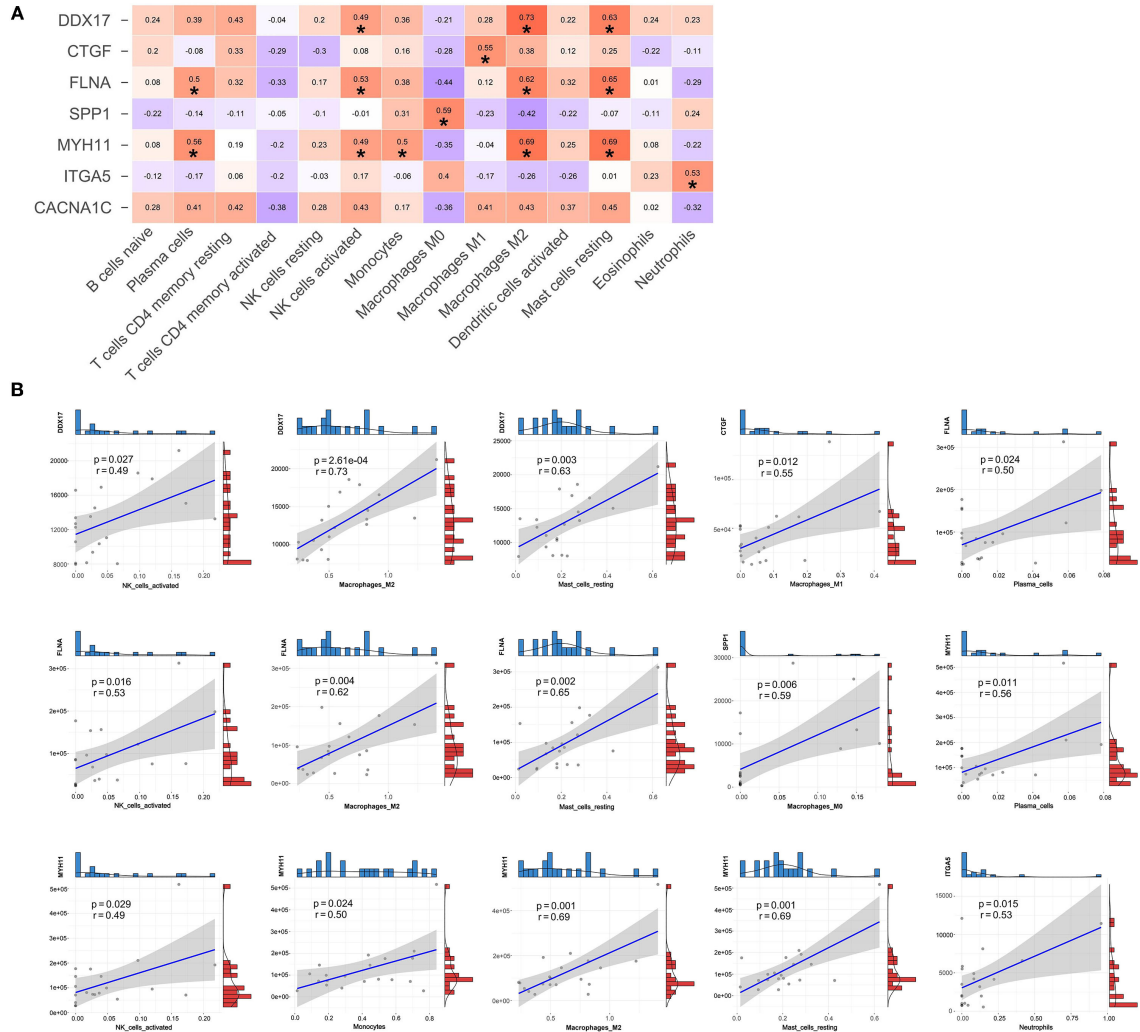
The relationships between hub genes and immune cells. **(A)** Correlation and significance of the relationships between hub genes and immune cells. **(B)** Scatter plot of significantly correlated between hub genes and immune cells.

In addition, we also performed the Pearson correlation analysis between hub genes and immune cells (Figure 9A). As shown in Figure 9B, DDX17 was correlated with NK cells activated (r = 0.49, *p* = 0.027), Macrophages M2 (r = 0.73, *p* = 2.61e-04) and Mast cells resting (r = 0.63, *p* = 0.003). CTGF was associated with Macrophages M1 (r = 0.55, *p* = 0.012). FLNA was correlated with Plasma cells (r = 0.50, *p* = 0.024), NK cells activated (r = 0.53, *p* = 0.016), Macrophages M2 (r = 0.62, *p* = 0.004) and Mast cells resting (r = 0.65, *p* = 0.002). SPP1 was related to Macrophages M0 (r = 0.59, *p* = 0.006). MYH11 was correlated with Plasma cells (r = 0.56, *p* = 0.011), NK cells activated (r = 0.49, *p* = 0.029), Monocytes (r = 0.50, *p* = 0.024), Macrophages M2 (r = 0.69, *p* = 0.001) and Mast cells resting (r = 0.69, *p* = 0.001). ITGA5 was correlated with Neutrophils (r = 0.53, *p* = 0.015).

## Discussion

The continuous and dynamic regulation of m6A has been shown to play a vital role in the physiological and pathological processes of cardiovascular diseases (CVDs) (20). However, few studies have reported the connection between m6A and AD so far (21). In this work, deeper data mining on AD was performed with bioinformatics tools, with GSE52093 and GSE153434 being used as the training set and test set, respectively. To ensure data reliability, a preliminary experiment was conducted in this study, and all samples from GSE52093 were selected for pre-analysis. In the formal research, ten samples with well-expression of DEGs found in pre-experiment were chosen to ensure the accurate analysis results. Meanwhile, a total of 279 differentially expressed m6A-related genes were screened, including 94 up-regulated and 185 down-regulated genes in AD. Then, the functions and pathways enriched by these genes were explored. In GO analysis, these genes were enriched into 12 GO terms, such as extracellular matrix organization, cytoplasm and protein binding. Upon KEGG pathway enrichment, the genes were mainly enriched into 2 pathways, namely vascular smooth muscle contraction and cGMP-PKG signaling pathway. By using the PPI network, 7 hub genes, namely, DDX17, CTGF, FLNA, SPP1, MYH11, ITGA5 and CACNA1C, were filtered. With regard to immune infiltration analysis, the level of B cells naive in AD samples was significantly reduced, while those of NK cells resting, Monocytes and Macrophages M0 increased in AD samples. Finally, the correlations between immune cells and between hub genes and immune cells were analyzed.

As indicated by GO functional annotation, the genes were mainly enriched in Cytoplasm and Protein binding. In the subsequent KEGG pathway enrichment analysis, the differentially expressed m6A-related genes were mainly enriched in cGMP-PKG signaling pathway and vascular smooth muscle contraction. Of them, the cGMP-PKG signaling pathway can regulate the vascular tone and is involved in smooth muscle-specific gene expression and phenotype (22, 23). It has been widely reported that the dysregulation of vascular smooth muscle contraction leads to predisposition to life-threatening AD (24, 25). The pathogenesis of AD is closely related to the phenotypic changes of SMCs, and the switch of pathological VSMC phenotype may trigger the occurrence and development of AD (26). We also sought to find out whether these differentially expressed m6A-related genes clustered at chromosomal location level, but unfortunately the degree of aggregation was not obvious. Moreover, there are few studies on the relationship between chr 1 and aortic diseases. The network of miRNA-mRNA relationships was established, MACF1, CHSY3, and TOR1AIP1 all interacted with two DEmiRs. But the association between these miRNA-mRNA relationship pairs and m6A methylation is currently unknown.

Here, based on the PPI network, 7 hub genes, namely, DDX17, CTGF, FLNA, SPP1, MYH11, ITGA5 and CACNA1C, were filtered. Among them, DDX17, an ATPase, can encode a DEAD box protein. The members of DEAD box family are implicated in a number of cellular processes that involve the alteration of RNA secondary structure (27). DDX5, another gene in the family, is required for maintaining the homeostasis of vascular SMCs (28). Noteworthily, DDX17 and DDX5 are the most closely related to each other in the DEAD box family. DDX17 can regulate smooth muscle cells together with DDX5, through regulating cell growth and division (29). As shown in a study, DDX17, a binding partner of cardiac physiological hypertrophy-associated regulator (CPhar), has the effect of regulating CPhar downstream factor ATF7 (activating transcription factor 7), and CPhar has cardioprotective effect (30). CTGF, also called CCN2, encodes a type of mitogen secreted by vascular endothelial cells (31). CTGF can mediate the adhesion, aggregation and migration of epithelial cells and SMCs (32). In terms of AD-related diseases, the up-regulated expression of CTGF is associated with atherogenesis, SMC apoptosis and aneurysm formation (33). Typically, CTGF is markedly over-expressed in human AS and can partially induce the apoptosis of SMCs. This effect may be important for the formation of atherosclerotic lesions (34). Recent studies on human vascular lesions have demonstrated that apoptosis is a prominent feature of both AS and restenosis, which are one of the causes leading to AD (35, 36). Therefore, AD may be further aggravated after CTGF induces SMC apoptosis. The hypomorphic and null mutations of FLNA can cause a wide spectrum of connective tissue and vascular anomalies (37). As reported in a study on hereditary large vessel diseases, FLNA's dysfunction disrupts the activities of transforming growth factor-β (TGF-β) signaling pathways, extracellular matrix and smooth muscle contractile apparatus, thus resulting in structural damage to AD (38). The TGF-β signaling is linked to the pathogenesis of CVDs like AS and cardiac fibrosis (39). An experiment has verified that FLNA is highly expressed in SMCs of aorta in non-AD samples, but down-regulated in the medial layer of the dissected aortas (40). Consistently, our bioinformatics research also came to the same result that FLNA was a down-regulated gene of AD. But the specific factors for FLNA down-regulation, especially the m6A RNA methylation modification pattern, remain unknown. Several transcript variants have been found for SPP1, whose polymorphisms are closely connected to the markers of carotid AS (41). A study discovers that SPP1 plays an important role in the physiological regulation of artery tone (42). But no existing study has found that SPP1 is directly related to AD. MYH11 gene is involved in vascular contractility and vascular wall stability (43, 44). For instance, it can decrease the proliferation and enhance the apoptosis of SMCs (45). Numerous studies on MYH11 and thoracic AD have been reported, and it is found that the heterozygous mutations of MYH11 are more susceptible to AD (7, 46). The up-regulation of ITGA5 may help to increase the synthesis of extracellular matrix (ECM) proteins and the dynamic remodeling of SMC-ECM interactions (47). A research on proteomic analysis draws a conclusion that ITGA-5 is a novel biomarker for the pathogenesis of AAD (48). But other studies on ITGA5 and AD are rarely reported. CACNA1C encodes an alpha-1 subunit of a calcium channel. Similarly, there are few studies on CACNA1C and AD. The dysregulation of CACNA1C may play a crucial role in the hypertension-induced endothelial dysfunction by affecting the calcium pathway (49). CACNA1C has been confirmed as a new susceptibility gene of calcific aortic valve stenosis (50).

The above seven genes are all modified by m6A methylation, and m6A may change the original functions of these genes to a great extent. Typically, m6A directs mRNAs to distinct fates by grouping them for differential processing, translation and decay in physiological processes such as cell differentiation and stress responses (51). Therefore, some functions of such hub genes are probably related to m6A modification, like being up-regulated in diseases, inducing smooth muscle apoptosis and mediating cell adhesion, aggregation and migration.

In this study, CIBERSORTx was applied in the immune infiltration analysis based on the GSE153434 dataset, which came to more accurate results. When analyzing the proportions of immune cells, the relative proportion data obtained from correlation analysis between immune cells were used. Therefore, the expression of hub genes and the relative proportions of immune cells must show a non-linear relationship. According to our immune infiltration results, the level of B cells naive in AD samples was significantly lower than that in normal samples, while those of NK cells resting, Monocytes and Macrophages M0 in AD were higher. Similarly, in a study on aorta B-cell immunity, Srikakulapu et al. discovered the highly territorialized B cell responses in arterial tertiary lymphoid organs compared with atherosclerotic lesions (52). Meanwhile, the research by Sara Rattik et al. also revealed that B-cells were associated with AD-related diseases, and that the transfer of B cells pulsed with the cholera toxin B subunit (CTB-p210) protected against AS (53). Besides, CTB-p210 might be useful to induce mucosal tolerance and reduce AS development (53). Monocytes play an important role in AS and display a considerable heterogeneity (54). The research by Li et al. found that specific depletion of monocytes and macrophages considerably inhibited the occurrence of AD and the infiltration of T lymphocytes and neutrophils (55). In this regard, monocytes and macrophages may work together in a certain relationship to promote AD. Furthermore, the data obtained by Lu et al. suggested that monocytes might play an important role in type B AAD (BAAD) (56). In terms of mortality, the lower lymphocyte-to-monocyte ratio may be independently associated with the mortality of type A AAD (AAAD) (57). Therefore, monocytes, which are related to both AAAD and BAAD, are the suitable candidates for further research. More research has focused on the relationship between macrophages and AD. The formation of AD is associated with aortic wall inflammation (58). Macrophages have pro-inflammatory (M1) and anti-inflammatory (M2) effects, which are involved in the development of AD and its complications (59). Angiotensin II has been identified as an important factor that stimulates macrophage activity (59), and IL-18 may increase the macrophage-induced apoptosis of SMCs. The study by Lian et al. revealed that macrophage metabolic reprogramming activated HIF-1α and subsequently promoted AD progression (60). Monocytes can differentiate into macrophages M0 (61), while macrophages M0 can be polarized into M1 and M2. In this study, macrophages M0 were positively correlated with monocytes. However, the mechanism underlying the different polarization directions in AD remains to be further illustrated. According to our results, macrophages M2, different from M1, might be related to Mast cells resting and Neutrophils in AD group. Mast cells can promote angiogenesis, recruit additional inflammatory cells, and stimulate vascular cell apoptosis. These activities are closely associated with the formation and development of AD (62). In addition, the accumulation of aortic neutrophils may be potentially related to the high incidence of AD (63).

Under certain conditions, CTGF induces an increase in M1 and a decrease in M2 macrophage markers for β-cell proliferation after injury (64). Our results in this work also showed that CTGF was positively correlated with M1 macrophages. The effects of DDX17 on macrophages have also been verified by experiments (65). However, the theoretical basis for the mechanism between m6A and immune cells in AD is still insufficient.

Until now, few studies have discovered the potential mechanism of action between m6A and AD. But it is strongly supported that m6A regulators (Writers, Readers and Erasers) can serve as the potential cardiovascular biomarkers. Some evidence suggests that aberrant m6A modifications affect the progression of CVDs, and the significantly elevated m6A methylation has been found in several CVDs (14). In this study, we tried to preliminarily explore the mechanism of m6A in AD. Based on our correlation analysis, we speculate that the amounts of some inflammatory cells may be related to the expression of the hub m6A gene, and the mechanism of m6A may also regulate the expression of certain genes in different inflammatory cells. Methyltransferase-like 3 (METTL3) is the well-known m6A methyltransferase, which functions in the reversible epi-transcriptomic modulation of m6A modification (66). The METTL3-mediated m6A methylation has been verified to be dynamic, and METTL3 silencing is reported to inhibit the apoptosis of cardiomyocytes subject to hypoxia or reoxygenation (67). In addition, METTL3 regulates inflammatory responses in chondrocytes and ECM synthesis (68). It is a direction worth studying about whether this mechanism also exists in METTL3 in other cells. The research by Qin et al. found that down-regulation of METTL3 attenuated SMC proliferation and migration. Simultaneously, the m6A modification level also remarkably decreased in SMCs (69). Therefore, m6A modification was related to AD, at least indirectly. However, the current research evidence is still insufficient, and the in-depth effects of m6A should be further studied.

To the best of our knowledge, this study is the first to investigate the mechanism underlying the combination of m6A and immune infiltration in AD. In terms of immune infiltration, different correlation analyses were performed, which represented one of the strengths of our research. Besides, verification using two datasets and joint analysis also improved our result accuracy. Nevertheless, certain limitations should be noted in this study. First, bioinformatics methods use data in the database, and the conclusions are almost completely affected by the original samples. These conclusions are not verified by experiments *in vivo* or *ex vivo*. In addition, the deep-level mechanism of m6A in AD has not yet been illustrated. In the future, it is desirable to verify our results based on *in vivo* and *in vitro* experiments, so as to discover more clear molecular mechanisms.

## Conclusion

Compared with normal samples, a large number of genes with m6A modification and differential expression are detected in AD. They are closely related to factors that cause AD such as smooth muscle cells. We not only identify immune cell infiltration in AD tissues, but also screen 7 hub genes regulated by m6A, namely, DDX17, CTGF, FLNA, SPP1, MYH11, ITGA5 and CACNA1C. These genes may become new biomarkers of AD, thus promoting the development of subsequent research and treatment.

## Data Availability Statement

Publicly available datasets were analyzed in this study. This data can be found at: GEO database GSE52093: https://www.ncbi.nlm.nih.gov/geo/query/acc.cgi?accGSE52093 GSE147027: https://www.ncbi.nlm.nih.gov/geo/query/acc.cgi?acc=GSE147027 GSE153434: https://www.ncbi.nlm.nih.gov/geo/query/acc.cgi?acc=GSE153434 SE92427: https://www.ncbi.nlm.nih.gov/geo/query/acc.cgi?acc=GSE92427.

## Author Contributions

FY and YH were involved in the conception and design of the study. FY and KL was responsible for visualization and article writing. CB provided scientific supervision. All authors reviewed and approved the final manuscript.

## Funding

This work was supported by the Fundamental Research Funds for the Central University (grant number: DUT19RC(3)076), the National Natural Science Foundation of China (grant number: 81600370), and the China Postdoctoral Science Foundation (grant number: 2018M640270) for YH.

## Conflict of Interest

The authors declare that the research was conducted in the absence of any commercial or financial relationships that could be construed as a potential conflict of interest.

## Publisher's Note

All claims expressed in this article are solely those of the authors and do not necessarily represent those of their affiliated organizations, or those of the publisher, the editors and the reviewers. Any product that may be evaluated in this article, or claim that may be made by its manufacturer, is not guaranteed or endorsed by the publisher.

## Supplementary Material

Supplementary File 1 The differentially expressed m6A-related genes in data pre-processing.

Supplementary File 2 Differentially expressed genes in GSE52093.

Supplementary File 3 Differentially methylated m6A genes in GSE147027.

Supplementary File 4 DEmiRs in GSE92427 and miRNA-mRNA pairs in miRDB, RTarBase, and Starbase database.

Supplementary File 5 GO and KEGG enrichment results.

Supplementary File 6 ROC monofactor analysis in GSE153434.
