# Supplementary figures and images for: Comprehensive Analysis of Key m6A Modification Related Genes and Immune Infiltrates in Human Aortic Dissection
Source: Front Cardiovasc Med. 2022 Mar 14;9:831561. doi: 10.3389/fcvm.2022.831561 (PMC8967178; doi:10.3389/fcvm.2022.831561)

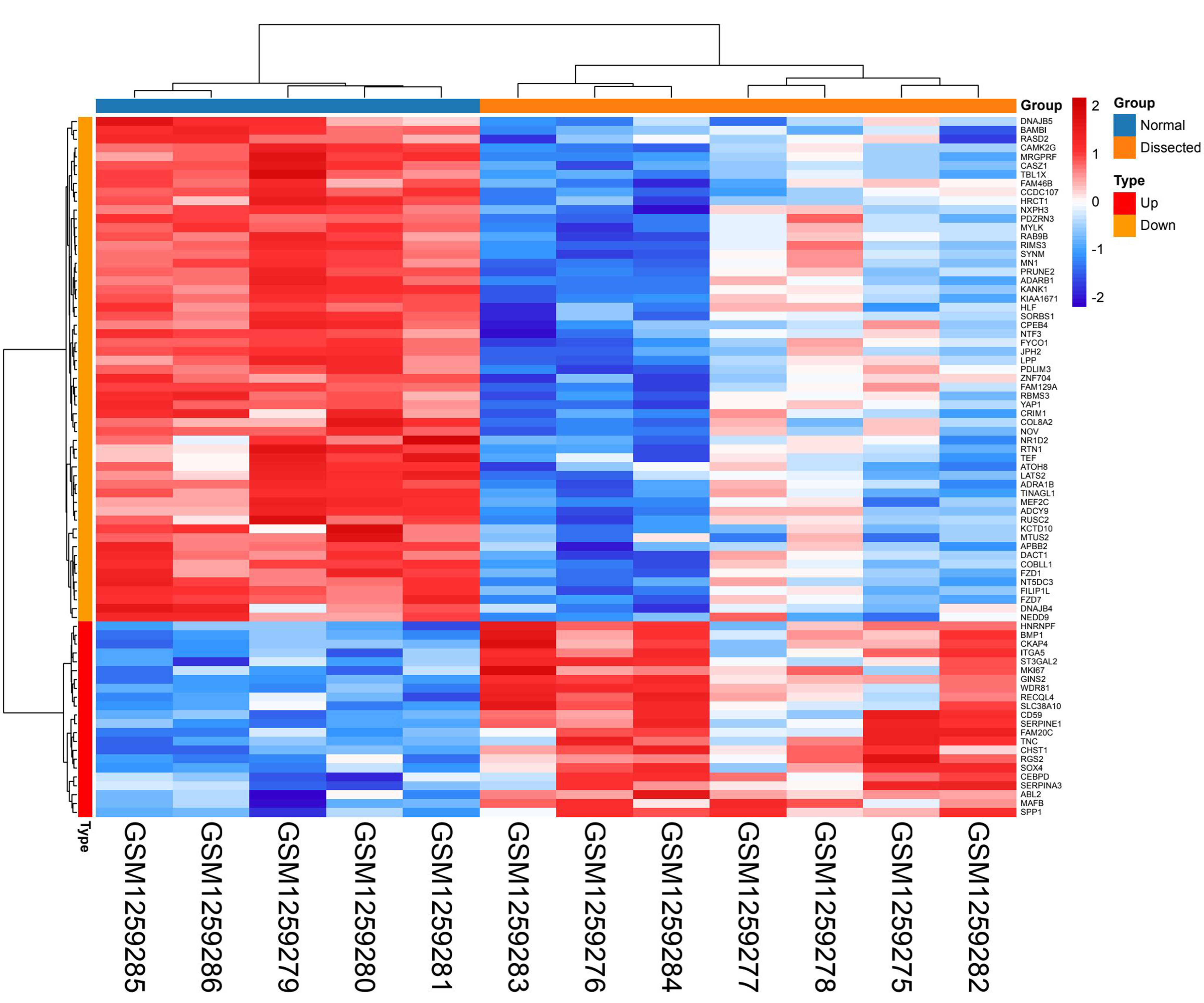

Supplement: Supplementary File 5 — GO and KEGG enrichment results. [file Image_1.JPEG]

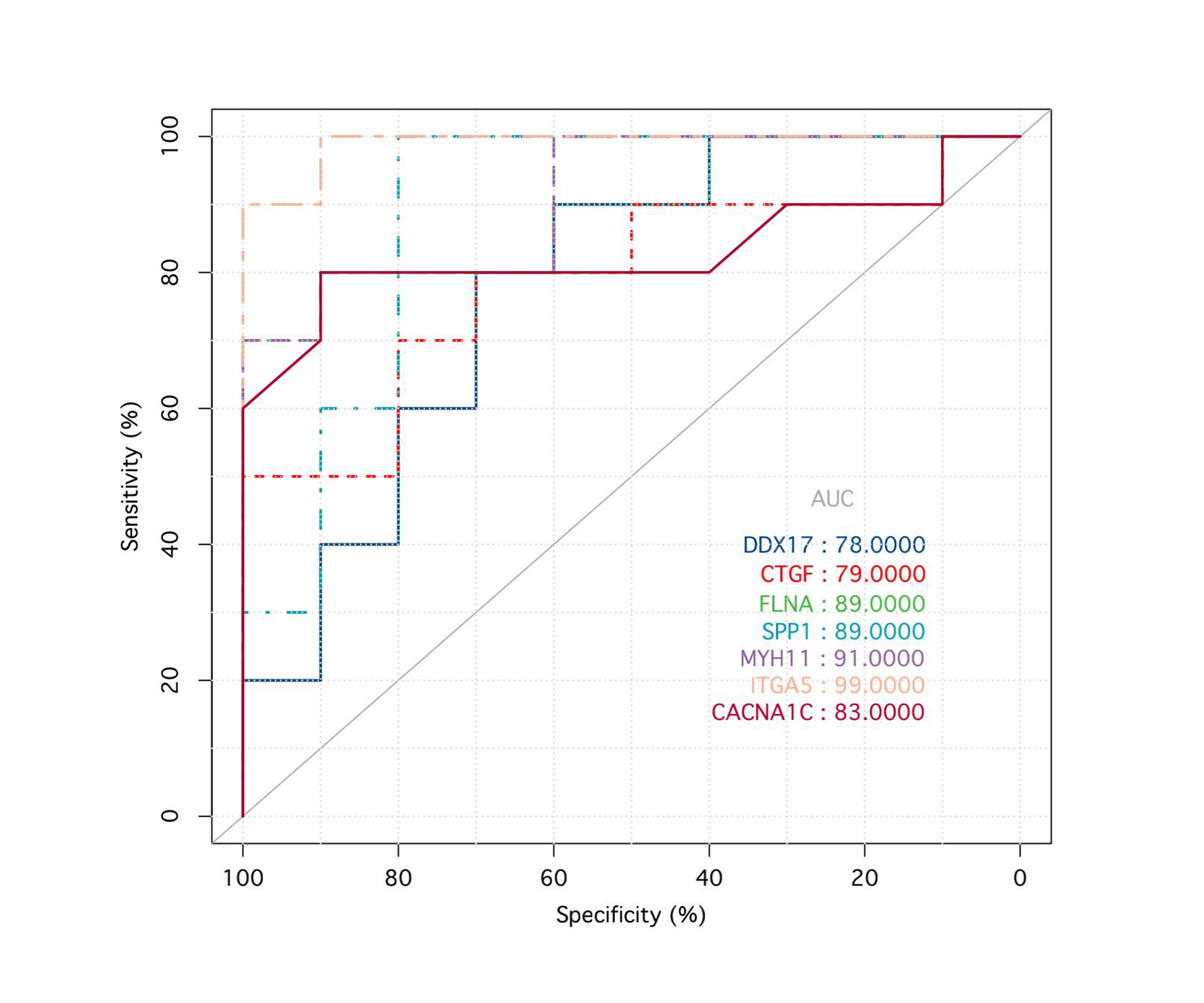

Supplement: Supplementary File 6 — ROC monofactor analysis in GSE153434. [file Image_2.JPEG]
